# Notes of a Case of Erysipelas

**Published:** 1864-04

**Authors:** D. W. Young

**Affiliations:** Aurora, Ill.


### ARTICLE XVIII.

### NOTES OF A CASE OF ERYSIPELAS.

By D. W. YOUNG, M.D., of Aurora, Ill.

Read before the Aurora City Medical Association. March 23d, 1864.

Gentlemen:—With your permission I will read the following notes of a case of erysipelas, that occurred in my practice recently. I do not present them with the expectation of offering anything new either in theory or practice, but for the purpose of adding further proof of the remarkable powers of muriated tincture of iron in arresting and curing erysipelas. I hold that it is only from the accumulated experience of practice that we can arrive at safe and certain results. In order to accumulate such experience, we must take notes and report our cases, otherwise no one but our individual selves will ever be benefited by our experience—medical literature will never be any the better for our having lived and practiced medicine. I regret that there is such an apparent apathy on the part of the older members of this association in reporting and discussing their important and interesting cases. I know they have them—treat them—and ought to report them. We tyros in the profession certainly need and are entitled to their experience—otherwise we must travel over the same uncertain ground over which they have travelled. Take our foreign text-books as a starting point, and then grope our way along the uncertain and mysterious avenues to experience. This is all wrong, gentlemen. You are unjust and illiberal. You are hiding your lights under a bushel. The good book pronounces that wicked. You should volunteer and report your cases; and thus give us your experience in managing and treating those grave diseases that are so liable to destroy our fellow-beings. In my judgment, there are too few medical associations; and those that do exist do too little in the way of reporting and recording their experience. A vast amount of information, and valuable unrecorded medical knowledge, is buried with almost every practitioner of medicine. This is lamentable—it is wrong—it is robbing medical science and humanity. Every practitioner of medicine should belong to some medical association, and there record and report his experience in the treatment and management of important diseases. Then would we accumulate the experience of the medical profession. But enough of this, gentlemen, excuse the digression. And now to the case of erysipelas:—

*Wednesday morning, March* 16, 1864.—I was called to see Peter Feischel, a stout and well-developed German boy, aged three and a-half years. I found him lying in bed comatose, and the left side of his face, head, and neck enormously swollen, very hard, red, hot, and shining. The swelling and inflammation extended down the neck and up over the cheek, face, and scalp, as far as the centre of the head. The left eye was closed, and the subcutaneous cellular tissue about it very much distended. There was an irregular abrupt elevated margin surrounding the disease, over the face and neck; while the scalp, as far as the disease extended, was very much swollen and puffy. The left side of the body, and the left arm and leg were enlarged and edematous—but neither red, hard, nor hot. The skin over this portion of the body was cool and livid. It had a peculiar look and feel. There was an emphysematous condition of the entire left side of the patient. Parotid and submaxillary glands on the left side largely swollen, red, and hot. Rolls his eyes constantly, and grates his teeth occasionally. Pulse 130 per minute, quick and feeble. Respiration slow and difficult. Skin cool and clammy. Bowels have not moved for several days. Passes his urine involuntarily.

Upon questioning his parents how long he had been sick, I obtained the following history of his case:—On the afternoon of the 6th of March, this little fellow and an older brother were seated together beside the stove with their playthings, had a boyish altercation, and the older brother pushed him so that he fell against the stove, and burned a small spot on the left side of his neck, just below the ear. It seemed so trivial that the parents paid no attention to it, and he soon resumed his sports and plays. He continued well, and played about the house the next and the succeeding days, as usual, so that they thought nothing further about the matter, until the next Sunday, just a week from the time of the accident, when he began to complain of being chilly, and his head aching. Then they noticed a small, bright red spot on his left cheek, just above where the burn had been, which was now healed. He complained considerably on Sunday afternoon; slept poorly that night; grew worse on Monday and Tuesday; and, finally, on Wednesday, I was called, and found him as above described. The condition of the little patient, when I first saw him, was unpromising indeed. I gave them a very doubtful prognosis, and ordered as follows:—℞. Soda bicarb. grs. vij., hydg. sub. mur. grs. v. Mix, and give immediately. Also, tinct. ferri chloridi, gtt. x. every hour: providing the stomach retains that amount without any unpleasant symptoms. Cover the inflamed surface with a warm bread and milk poultice. Keep the room dark and quiet as possible.

*Thursday morning, March 17th, 9 o'clock A.M.*—Cathartic has operated twice thoroughly. Patient is still comatose. The emphysematous appearance has disappeared from the left side, outside of the erysipelatous swelling. Swelling about the face, neck, and scalp about the same; I think it is not quite so red, hard, or hot. Respiration better; skin warmer; pulse 120 per minute, fuller and softer. Secretions still passed involuntarily. Patient is more restless. Pupil of left eye largely dilated and stationary; still rolls his eyes and grates his teeth. Ordered as follows:—Continue the iron every hour, and increase one drop every second dose, until xv gtt. are given every hour, providing he retains it well. Continue the bread and milk poultices, change them often. Also give, ℞. Quinia sulph. grs. j., pulv. Doveri, grs. ij., every three hours. Feed him chicken broth or beef tea liberally.

*Friday morning, March 18th, 9 o'clock A.M.*—Patient still comatose. Swelling about the left side of head less. Erysipelas has extended considerably on the right side, but is not so intense. The iron is certainly modifying the disease decidedly. He retains the fifteen drop doses without any trouble. Pupil of left eye still dilated. Does not roll his eyes nor grate his teeth this morning. Sleeps more quietly. Color and temperature of surface about the same. Discharges still involuntary. Respiration free and easy. Pulse 122 per minute, slower and soft. Continued all the treatment as at last visit, only increase the tinct. ferri chloride one drop every second hour, until he takes gtt. xx. every hour, if he retains it well.

*Saturday moming, March 19th, 9 o'clock A.M.*—Patient still comatose. Swelling on left side disappearing rapidly, and desquamation commencing. Disease has extended a little on the right side, but is fading. He opens his left eye, and the pupil is not dilated as much, and begins to respond to light. He bears the medicines and nourishment well. Called for a drink of water in the night, which is the first word he has spoken since I commenced the treatment. Feces and urine still passed without any apparent knowledge of the patient. Skin warm and moist; pulse 118 per minute. The cellular tissue about the right eye is becoming considerably distended, and the eye partially closed. General aspect of patient bad. Head and face are enormously swollen, and he has a bad look—almost frightful. Continue same treatment. Increase the muriated tinture of iron one drop every second dose, until gtt. xxv. are taken every hour. Continue quinine and Doveri as before. Feed him liberally.

*Sunday morning, March 20th, 9 o'clock A.M.*—Patient is better. Retains medicines and nourishment well. Passed a good day and night since last visit. Erysipelas has not extended any and is fading. There are two small spots, one on right cheek and one on nose, that look angry and bad. Made a solution of nitrate of silver and touched them lightly. He is now semi-comatose. Passes his secretions involuntarily; but rouses when spoken to and attempts to speak. Swelling about the face disappearing. Opens both eyes partially, and the pupils respond to light. There is no rolling of the head nor grating of the teeth. Skin warm and moist; pulse 98 per minute, full and soft. I feel encouraged about him; think he may recover; but urged the greatest care and caution in his nursing and management. Ordered that the room be kept dark and perfectly quiet. Continued all the medicines and applications as at last visit.

*Monday morning, March* 21*st*, 9 *o'clock A.M.*—Found patient much improved. The swelling is nearly gone and the erysipelas *played out.* Retained the medicines and nourishment well. The xxv. gtt. doses of the muriated tinct. of iron every hour have been taken regularly and retained without any nausea or vomiting. He opens both eyes well, and is rational. Begs for his clothes and to be dressed. Pulse 94 per minute and regular. Bowels open, urine free. Skin warm, soft, and moist. Desquamation free and extensive. Hair falling out rapidly. Begs for food constantly. Continue the quinine and Dover's powder as before. Reduce the iron to xx gtt. once in two hours. Discontinue the poultices.

*Tuesday morning, March* 22*d*, 9 *o'clock A.M.*—Found patient bolstered up in bed with his clothes on and playthings around him. Swelling has pretty much disappeared and so has the erysipelas. Desquamation extensive. Hair falling off profusely. Altogether he presents a decidedly shabby appearance about the head and face. Bowels act well; urine free and very high colored. Pulse 92 per minute, slow and soft. Slept very quietly all last night. Appetite craving. Stop the quinine and Dover's powder. Reduce the iron to x gtt. once in four hours. Feed him liberally with animal broths, but not too much at a time. Keep him quiet and from scratching his face —to prevent this put gloves on his hands.

*Wednesday morning, March* 23*d*, 11 *o'clock A.M.*—Found patient improving nicely. Slept well all night. Pulse 90 and regular. Everything looks well. Bowels act well; urine free and not so high colored. Reduce the tinct. ferri to v gtt. every six hours, for to-day, then discontinue it entirely. Cautioned them to keep him warm, well clothed, and nourished. Discharged him convalescent.

*Remarks.*—The principal points of interest in this case are, first, the severity and persistence of the disease; second, the large doses of muriated tincture of iron administered. It will be noticed that xxv gtt. were given every hour for two days and nights. This, in a boy three and a-half years old, I admit is heroic medication. The case was an extensive one, and demanded, I thought, extreme measures. In treating the case I adopted the same rule that I am in the habit of following in treating all disease. I select my remedies and then push them until I see some effect from them. Here I commenced with x gtt. every hour, continued that dose for two days; it produced no unpleasant symptoms and but little if any amelioration of the disease. Then I increased one drop every second dose until xv gtt. were given every hour; continued that dose two days and nights. It produced no unpleasant effects, and only seemed to hold the disease stationary. Then I increased one drop again every second hour until xx gtt. were given every hour. This dose was also continued two days and nights, produced no unpleasant symptoms, but a marked improvement in the disease. Consciousness began to return. Pulse became slower and fuller. At the end of the second day, the dose was again increased one drop every second dose until xxv gtt. was given every hour. This dose was also retained equally well; I could discover no unpleasant symptoms from it; it controlled the disease immediately, and the patient improved rapidly. I obtained the desired result, and therefore had no occasion to increase the dose further. Possibly there may still be some doubt in the minds of some of the members of our profession as regards the antidotal powers of muriated tincture of iron in erysipelas. There are none in mine. I have used it a long time extensively, both in civil and military practice. I am a convert—a full believer.

